# RIPK4 Is an Immune Regulating-Associated Biomarker for Ovarian Cancer and Possesses Generalization Value in Pan-Cancer

**DOI:** 10.1155/2022/7599098

**Published:** 2022-03-09

**Authors:** Cui Liao, Yi-xia Zhao, Wei-di Han, Nian-yu Lai

**Affiliations:** ^1^Chongqing Key Laboratory of Translational Research for Cancer Metastasis and Individualized Treatment, Chongqing University Cancer Hospital, Shapingba District, Chongqing 400030, China; ^2^Department of Pharmacy, The General Hospital of Northern Theater Command of Chinese PLA, Shenyang, 110016 Liaoning Province, China

## Abstract

Ovarian cancer (OC) is the most lethal gynecologic cancer. Many studies have reported that RIPK4 (receptor interacting serine/threonine kinase 4) displayed a dysregulated level in many types of tumors. However, its expressions and functions in OC were rarely reported. The levels of RIPK4 were detected in OC and nontumor specimens using TCGA and GEO datasets. The prognostic values of RIPK4 in patients were determined using Kaplan-Meier methods and Kaplan-Meier assays. GO assays and KEGG pathway assays were carried out for functional enrichments. CIBERSORT was applied for estimating the fractions of immune cell types. Finally, RIPK4 was validated in pan-cancer. In this study, our group found that RIPK4 exhibited a higher level of RIPK4 in OC specimens than nontumor specimens. Survival studies revealed that patients with high RIPK4 expressions showed a shorter overall survival than those with low RIPK4 expression. Multivariate assays further confirmed that RIPK4 expression was an independent prognostic element for OC. KEGG pathway analysis displayed that the dysregulated genes in specimens with high RIPK4 expressions were enriched in focal adhesion, proteoglycans in cancer, central carbon metabolism in cancer, and insulin secretion. Correlation analyses showed that several TICs were positively correlated with RIPK4 expression. The pan-cancer validation results showed that RIPK4 was associated with survival in five tumors. Overall, our findings suggested RIPK4 as a prognostic marker in OC.

## 1. Introduction

Ovarian cancer (OC) is a significant cause of death from gynecologic cancer [1]. There are >15,000 patients who died of OC each year in the United States [2]. Many patients with early stages do not display specific symptoms, and effective screening methods are limited in clinical practice, which results in >65% of OC patients with advanced stages (FIGO stage III or IV) were diagnosed [3, 4]. To date, chemotherapy and surgery remain the standard treatments for OC. Although new research results for OC progression are constantly emerging, advanced-stage diseases do not tend to respond well to cytotoxic chemotherapy and are associated with a poor outcome, which encourage researchers to explore sensitive biomarkers and the potential mechanisms involved in OC progression and metastasis for the improvements of clinical outcome of OC patients [5, 6].

As a member of the RIPK family, receptor-interacting protein kinase 4 (RIPK4) was originally identified as a regulator involved in protein kinase C (PKC) via yeast two-hybrid-based screen [7]. It has been demonstrated that RIPK4 mutations may result in popliteal pterygium syndromes [8]. Previously, many researches focused mainly on keratinocyte proliferation and differentiation [9, 10]. Recently, a large number of studies have reported that RIPK4 expressions were distinctly dysregulated in many types of tumors, such as nasopharyngeal carcinoma, osteosarcoma, and bladder urothelial carcinoma cell [11–13]. In addition, previous in vivo assays indicated that knockdown of RIPK4 suppressed tumor growth [14]. On the other hand, RIPK4 was demonstrated to be involved in the activity of NF-*κ*B signals [15, 16]. The above findings indicated RIPK4 as a tumor promotor, and the dysregulation of NF-*κ*B pathway was confirmed to be involved in progression of many neoplasms [17, 18]. However, the biological function of RIPK4 in OC remains unclear. In this study, we aimed to explore the prognostic value and tumor immunity relevance of RIPK4 in OC patients.

## 2. Materials and Methods

### 2.1. Data Collection

Pan-cancer data, including clinical data and gene expressions were downloaded from TCGA datasets. Survival data of TCGA cohorts were obtained from the integrated TCGA pan-cancer resources. Abbreviations of all types of tumors are exhibited in Table S1. NCBI-GEO is a free public database of microarray datasets and we downloaded sequencing data of GSE36668, which include 8 OC tissues and 4 nontumor tissues [19]. Microarray data of GSE36668 were all on account of GPL570 platforms ([HG-U133_Plus_2] Affymetrix Human Genome U133 Plus 2.0 Array).

### 2.2. Possible Values of RIPK4 in Survival Assays

We divided patients into high or low group based on the mean expression of RIPK4. Kaplan-Meier methods were carried out for survival curves, and log-rank was conducted to determine the distinct significance by the use of R packages survival and survminer in R 3.6.0.

### 2.3. Gene Ontology (GO) and Kyoto Encyclopedia of Genes and Genomes (KEGG) Enrichment Assays of the Aberrantly Expressed Genes

To delve in-depth into the promising functions and pathways of the common aberrantly expressed mRNAs, we carried out GO and KEGG enrichment assays by the use of the “clusterProfiler” package in R with a statistical threshold of *p* < 0.05.

### 2.4. Immune Infiltration

(CIBERSORT) algorithm (http://cibersort.stanford.edu/) was applied to display the proportion of 22 kinds of immune cells in each OC sample from TCGA datasets (16). 1,000 simulations were exhibited, and *p* < 0.05 was distinct.

### 2.5. Statistical Analysis

All statistical analyses were performed with R software 3.5.3. The difference between two groups was analyzed by Student's *t*-test. For survival analysis, the Kaplan-Meier curve was generated with the log-rank test. Univariate and multivariate assays were carried out to examine the HR of prognostic factors. The receiver operating characteristic (ROC) curve was applied to examine the accuracy of RIPK4 expressions by comparing the area under the curves (AUCs). Statistical significance was set at a probability value of *p* < .05.

## 3. Results

### 3.1. Increased Expressions of RIPK4 in Clinical OC Specimens

**Figure 1:**
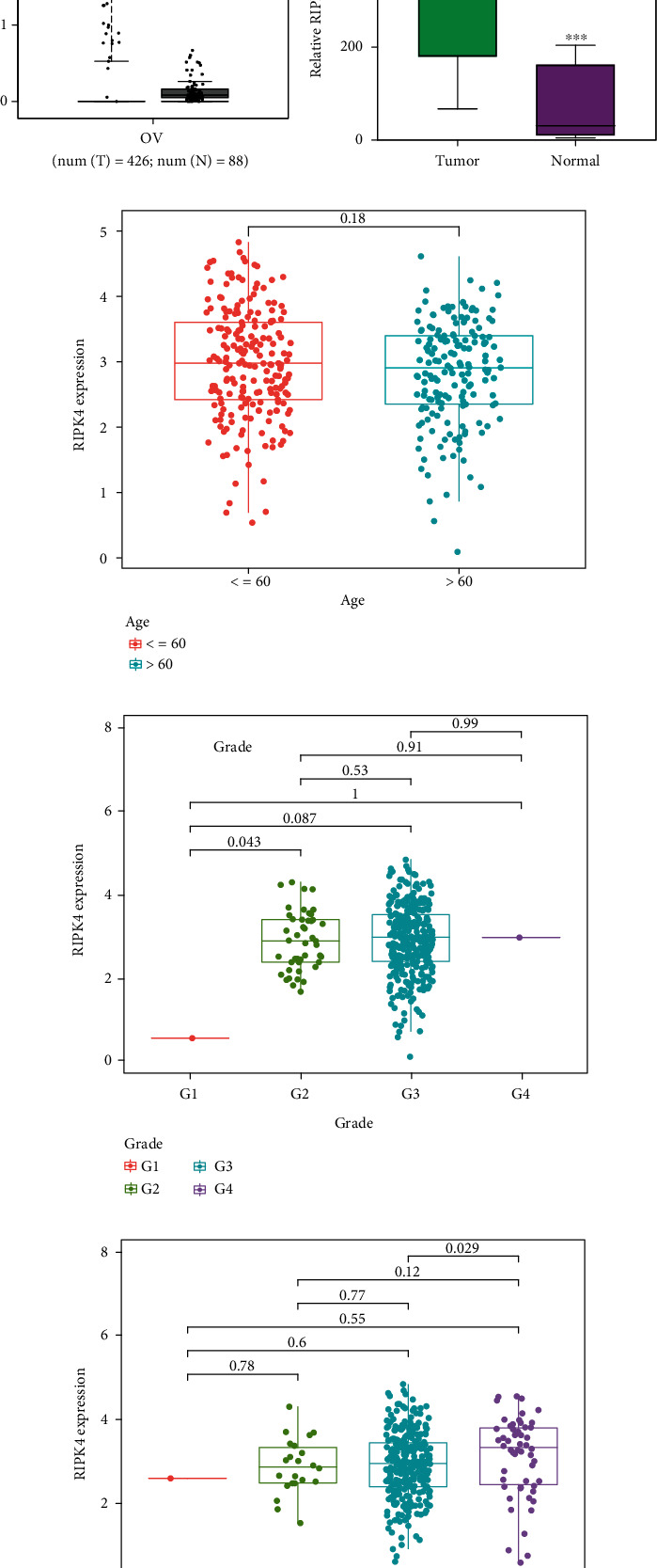
Analysis for the expression and clinical significance of RIPK4 in OC. (a) The distinct upregulation of RIPK4 in OC from TCGA datasets. (b) Differential expression of RIPK4 in OC from GSE36668. (c–e) The relationship between TBX5-AS1 and clinical features; (c) age. (d)grade. (e) stage. ^∗^*p* < 0.05 and ^∗∗∗^*p* < 0.001.

To delve into the possible functions of RIPK4 in OC progression, we applied GEPIA to analyze its expressions in OC specimens and nontumor specimens. As displayed in Figure 1(a), we observed that RIPK4 levels were distinctly increased in OC specimens compared with nontumor specimens. Moreover, the results of GSE36668 also identified RIPK4 as an overexpressed gene in OC (Figure 1(b)). Then, we analyzed its clinical association with several features, finding that the expressions of RIPK4 were not associated with age and grade in OC patients (Figures 1(c) and 1(d)). However, we found that OC specimens with sage IV exhibited a distinctly higher level of RIPK4 than those with early stages (Figure 1(e)).

### 3.2. RIPK4 Overexpression Is Poor Prognostic Factor for OC Patients

**Figure 2:**
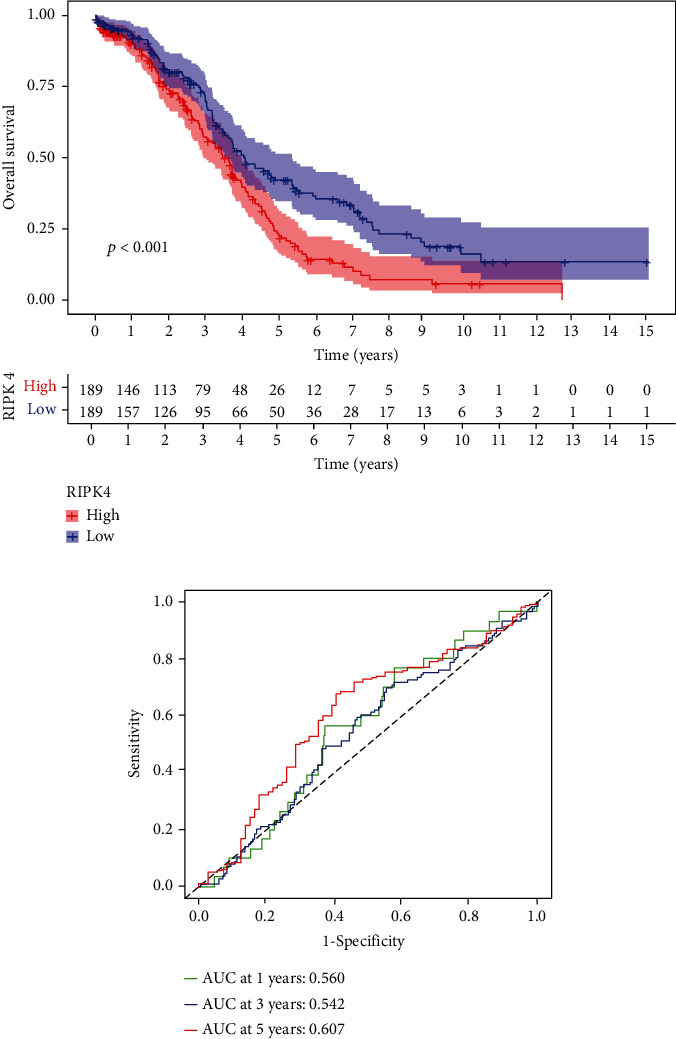
(a) Kaplan-Meier curves for the OS of patients in the high RIPK4 expression group and low RIPK4 expression group in OC patients. (b) The ROC curves represented the discrimination of the models measured by the C-index.

**Figure 3:**
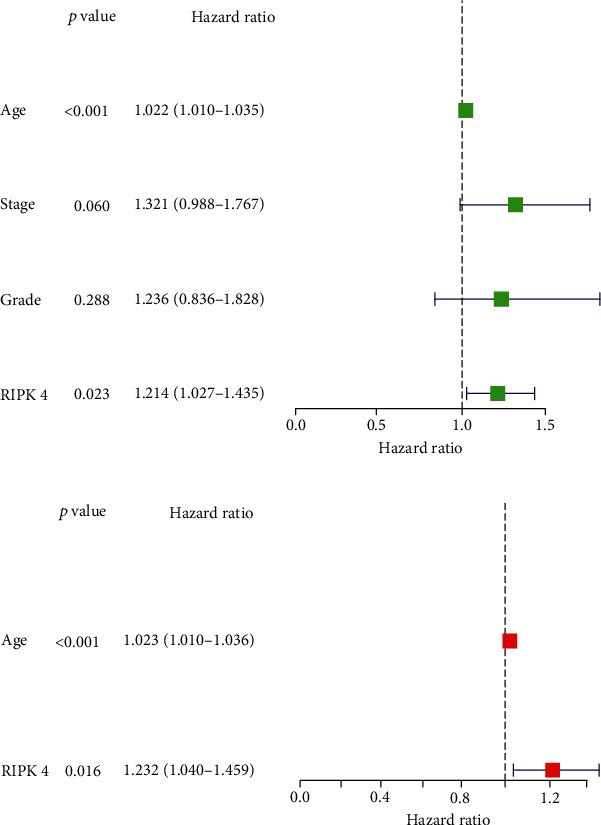
(a) Univariate analysis of RIPK4. (b) Multivariate analysis of RIPK4.

Then, our group performed survival analysis. Kaplan-Meier assays demonstrated that high RIPK4 expression predicted a shorter OS (Figure 2(a)). However, the time-dependent ROC curves did not further demonstrate the predictive accuracy of RIPK4 expression in long-term survivals of OC patients (Figure 2(b)). Next, univariate and multivariate assays were applied to further determine the potential of RIPK4 expressions and clinicopathological features used as novel biomarkers for OC patients. As shown in Figure 3(a), according to univariate assays, age and RIPK4 expression were statistically significant prognostic factors. More importantly, the results of multivariate assays confirmed that RIPK4 expression was an independent prognostic factor (Figure 3(b)).

### 3.3. GO and KEGG Analysis

**Figure 4:**
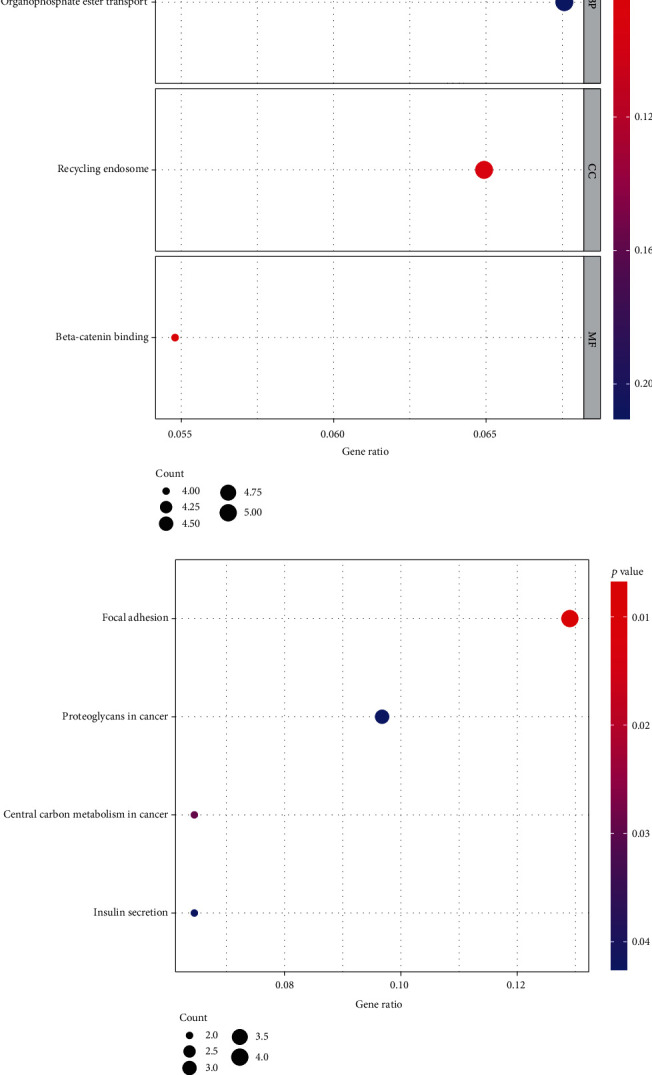
(a) Significantly enriched Gene ontology (GO) terms of dysregulated genes in OC. (b) Significant Kyoto Encyclopedia of Genes and Genomes (KEGG) pathway terms of dysregulated genes in OC.

To further understand the potential role of RIPK4 in OC, GO and KEGG analyses were performed on the dysregulated genes between specimens with high RIPK4 expression group and specimens with low RIPK4 expression group. The results revealed that these genes were mainly involved in organophosphate ester transport, recycling endosome, and beta-catenin binding (Figure 4(a)). KEGG pathway analysis displayed that the dysregulated genes were enriched in focal adhesion, proteoglycans in cancer, central carbon metabolism in cancer, and insulin secretion (Figure 4(b)).

### 3.4. Associations of RIPK4 with the Proportion of Tumor-Infiltrating Immune Cells (TICs)

**Figure 5:**
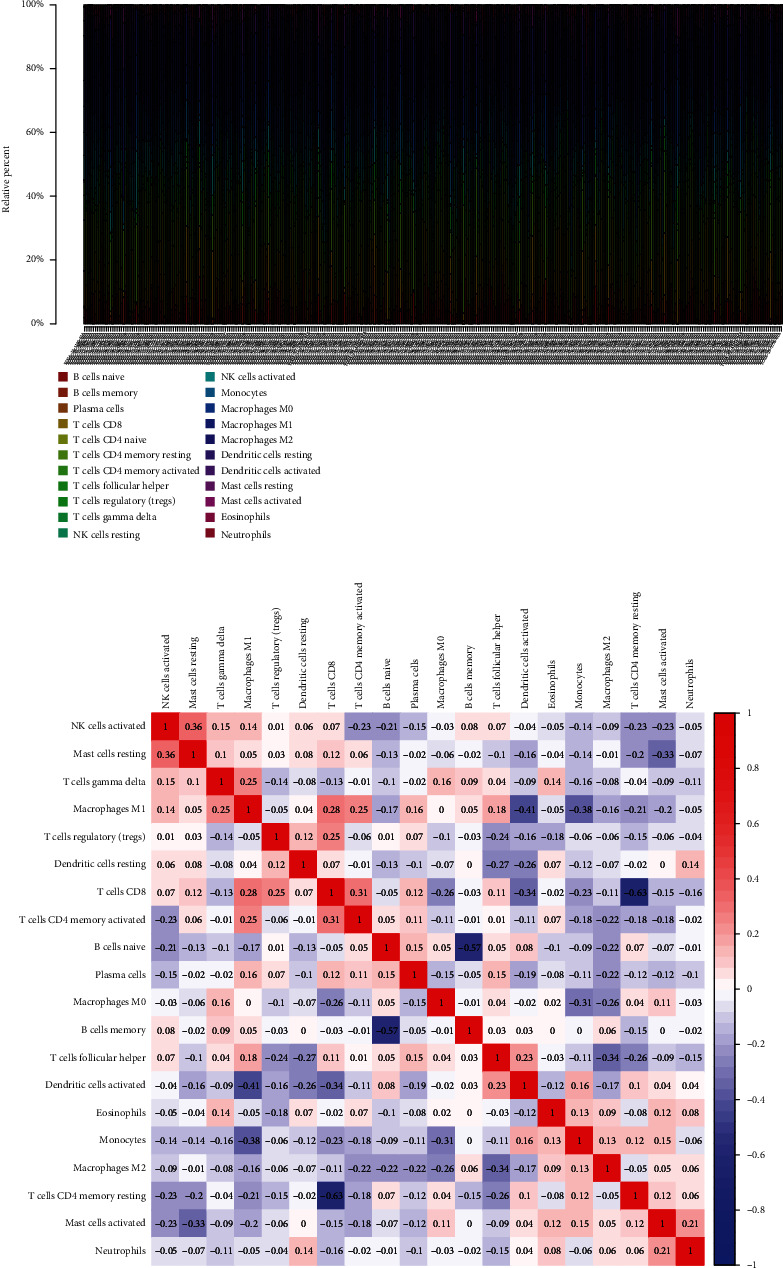
TIC profile in OC specimens and correlation assays. (a) The proportion of 22 types of immune cells in all OC samples. (b) Heatmap showing the correlation between 21 kinds of TICs.

**Figure 6:**
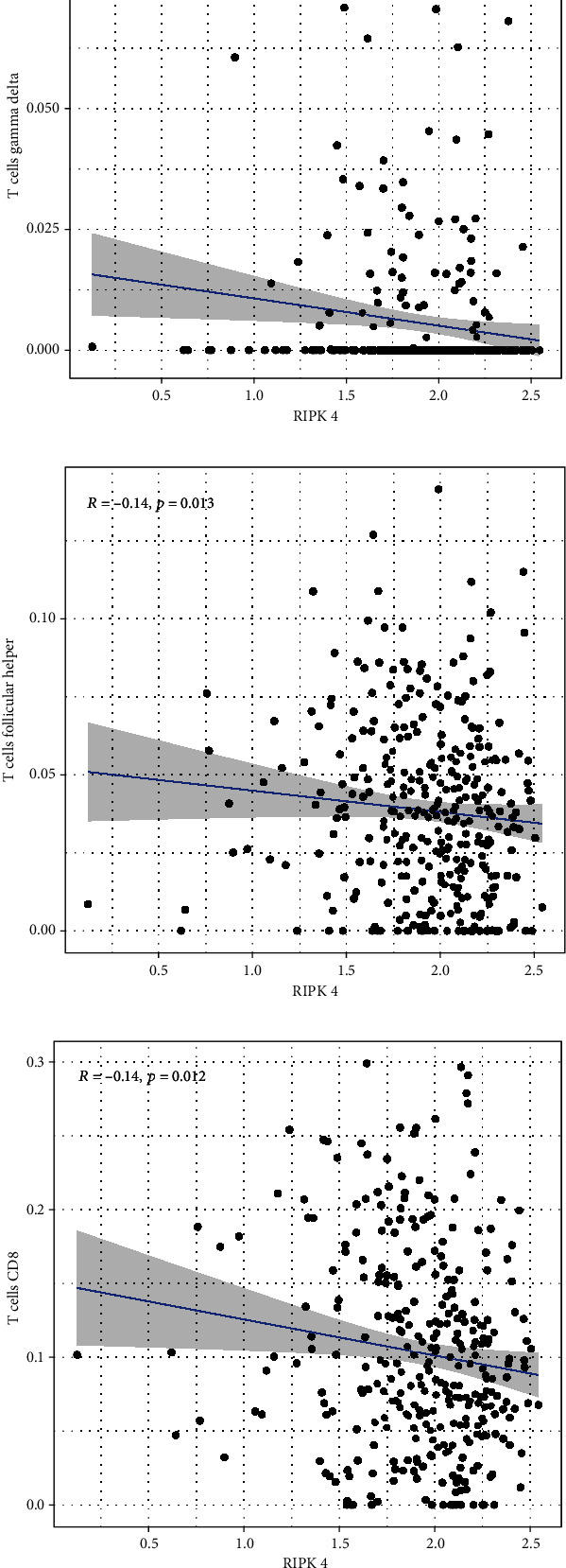
Correlation between RIPK4 level and (a) monocytes, (b) T cell CD4 memory resting, (c) T cell gamma delta. (d) T cell follicular helper, (e) T cell CD8, (f) T cell CD4 memory activated, and (g) Macrophages M1.

To explore the associations of RIPK4 expressions with the immune microenvironment, we carried out the CIBERSORT algorithm. As presented in Figures 5(a) and 5(b), 21 kinds of immune cell profiles in OC samples were shown. We observed that two kinds of TICs were positively correlated with RIPK4 expressions, including monocytes and T cell CD4 memory resting (Figures 6(a) and 6(b)). In addition, five kinds of TICs were negatively related to RIPK4 expression, including T cell gamma delta, T cell follicular helper, T cell CD8, T cell CD4 memory activated, and macrophages M1 (Figures 6(c)–6(g)).

### 3.5. Pan-Cancer Verification

**Figure 7:**
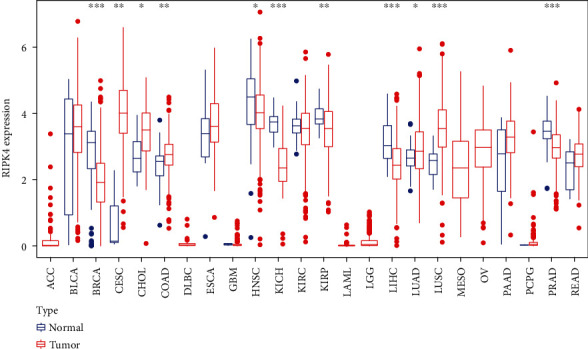
The expression level of RIPK4 in TCGA samples. ^∗^*p* < 0.05, ^∗∗^*p* < 0.01, and ^∗∗∗^*p* < 0.001.

**Figure 8:**
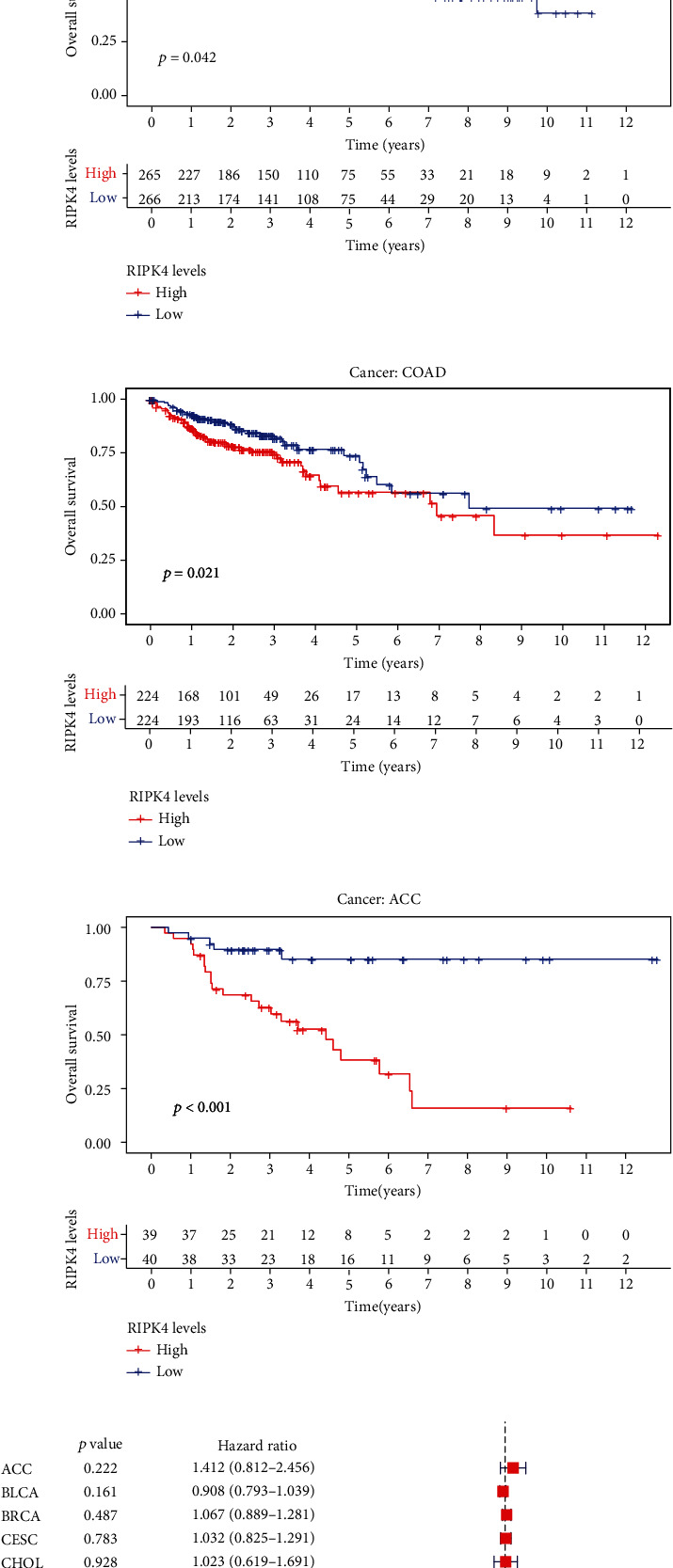
(a–d) Kaplan-Meier survival curves of overall survival for RIPK4 in pan-cancer (*p* < 0.05). (e) Multivariate analysis was performed to determine the prognostic value of RIPK4 in OS in pan-cancer.

**Figure 9:**
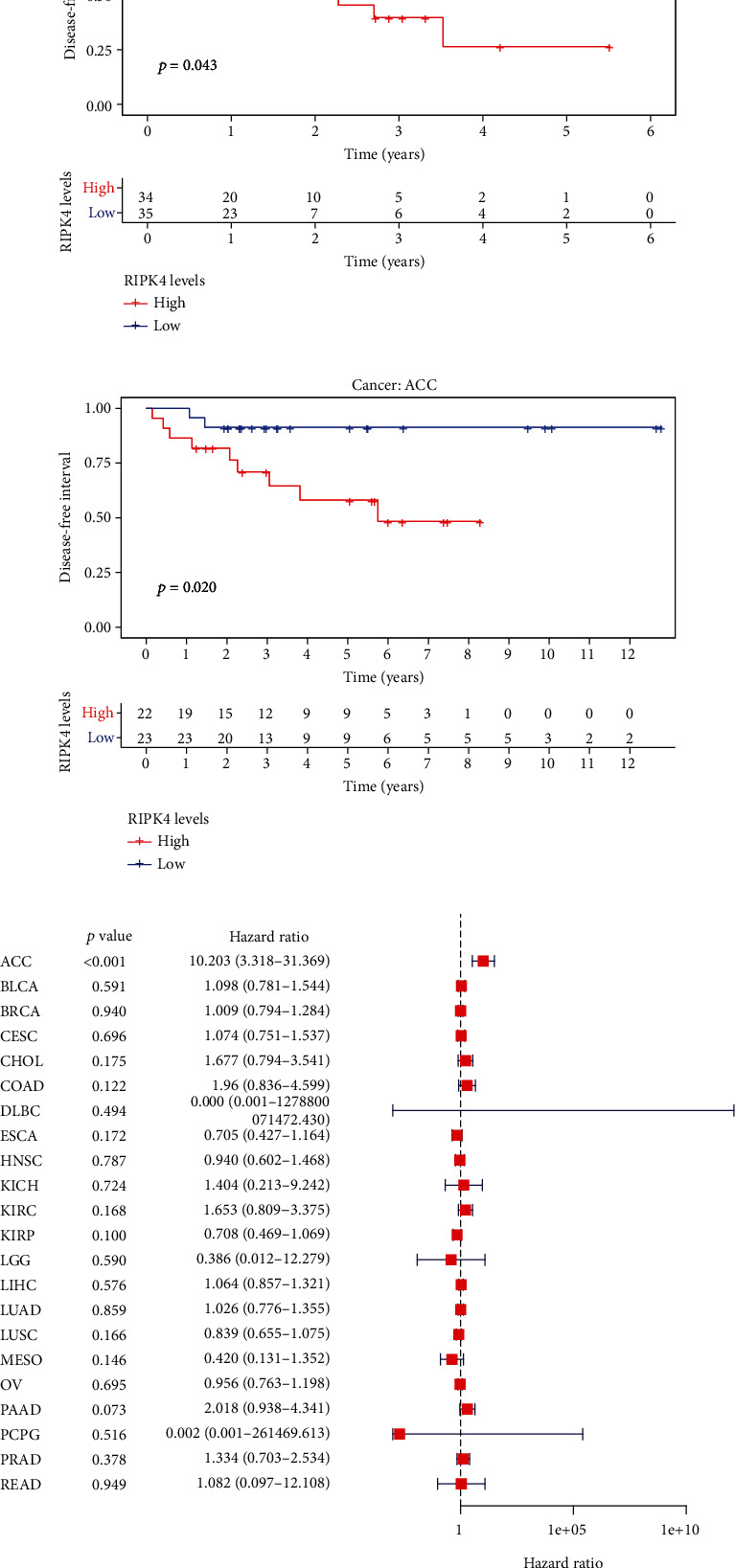
(a and b) Kaplan-Meier survival curves of disease-free interval for RIPK4 in pan-cancer (*p* < 0.05). (c) Multivariate analysis for RIPK4 expression in disease-free interval in pan-cancer.

**Figure 10:**
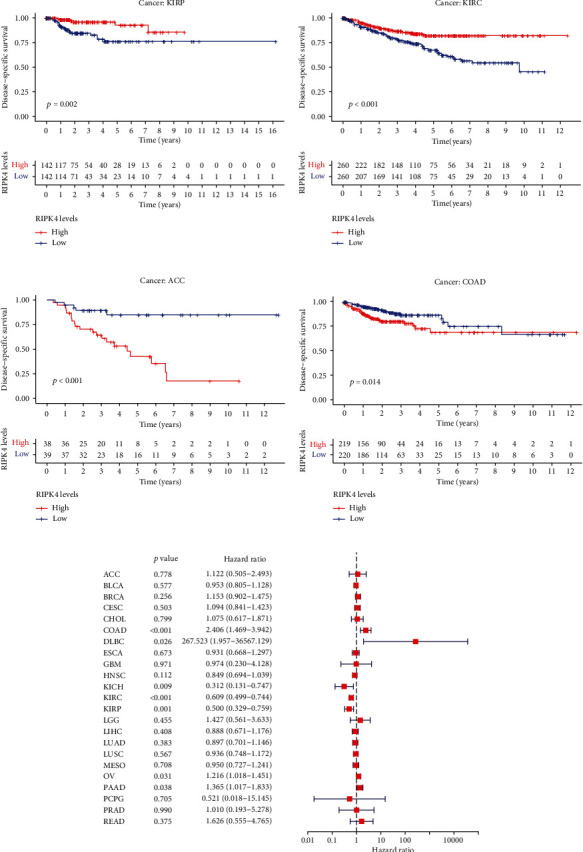
(a–d) Kaplan-Meier survival curves of disease-specific survival for RIPK4 in pan-cancer (*p* < 0.05). (e) Multivariate analysis for RIPK4 expression in disease-specific survival in pan-cancer.

**Figure 11:**
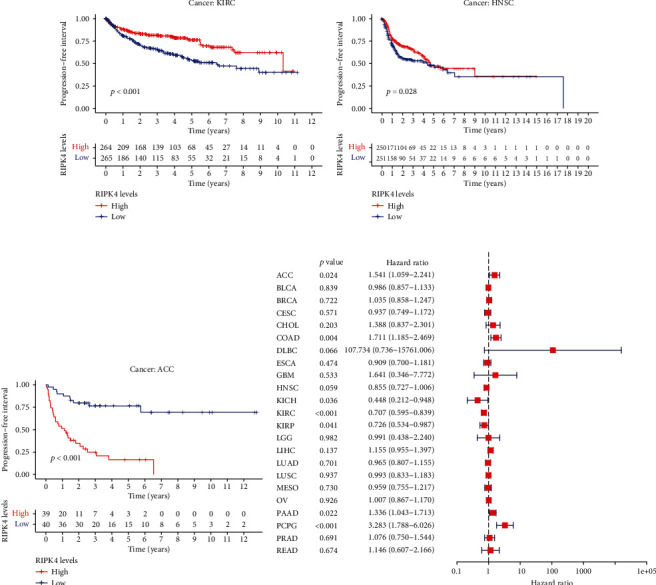
(a–c) Kaplan-Meier survival curves of progression-free interval for RIPK4 in pan-cancer (*p* < 0.05). (d) Multivariate analysis for RIPK4 expression in disease-specific survival in pan-cancer.

The expressing pattern of RIPK4 in pan-cancer is shown in Figure 7. To further examine the possible abilities of RIPK4 as a potential biomarker in pan-cancer, we conducted survival assays. The data indicated that RIPK4 was associated with OS in four tumors, namely, KIRP, KIRC, COAD, and ACC (Figures 8(a)–8(d)). Multivariate analyses confirmed RIPK4 as an independent prognostic factor of OS of patients with KICH, KIRC, KIRP, and PAAD (Figure 8(e)). We also observed that RIPK4 was related to disease-free interval in two tumors, including PAAD and ACC (Figures 9(a)–9(c)). Moreover, RIPK4 was related to disease-specific survival in four tumors, including KIRP, KIRC, ACC, and COAD (Figures 10(a)–10(e)). Finally, RIPK4 was associated with progression-free interval in three tumors, including KIRC, HNSC, and ACC (Figures 11(a)–11(d)).

## 4. Discussion

OC is a salient public health concern. High-grade malignancy, early metastasis, rapid infiltrating growth, and poor clinical outcome are imperative characteristics of OC [20]. More and more evidences have demonstrated that several types of markers are able to predict long-term survivals of different tumors, such as OC [21, 22]. In recent years, many researches have provided some prognostic and diagnostic markers for OC, such as functional mRNAs, ncRNAs, and DNA methylation [23, 24]. Among them, some functional genes involved in OC progression offer a new direction and have attracted much attention.

Recently, several studies reported the roles of RIPK4 in tumor progression. For instance, RIPK4 mutations were observed in several squamous cell carcinomas [25, 26]. The cutaneous squamous cell carcinomas data deposited in TCGA and the assays of metastatic squamous cell carcinomas reported a similar high rate of RIPK4 mutagenesis. Gong et al. reported that RIPK4 expression was upregulated in nasopharyngeal carcinoma, and its silence exhibited a suppressor effect on the proliferation and metastasis of tumor cells via activating NF-*κ*B signaling [11]. In bladder urothelial carcinoma, RIPK4 was shown to exhibit a high level and its silence inhibited the tumor growth and migration of bladder urothelial carcinoma cells via modulating NF-*κ*B pathway [13]. Previously, a study by Yi et al. firstly reported the oncogenic roles of RIPK4 in ovarian cancer [27]. However, the clinical significance of RIPK4 was rarely reported. In this research, we analyzed TCGA and GSE36668 datasets and observed that RIPK4 expressions were distinctly increased in OC specimens compared with nontumor specimens. Clinical studies revealed that cases with high RIPK4 levels exhibited a shorter OS. Moreover, KEGG assays indicated that RIPK4 expression may be associated with focal adhesion, proteoglycans in cancer, central carbon metabolism in cancer, and insulin secretion. To further explore the prognostic value of RIPK4 in tumors, we performed pan-cancer assays and observed that RIPK4 expression was associated with KIRP, KIRC, PAAD, COAD, and ACC. Overall, our findings suggested RIPK4 as an oncogene in OC progression, which was consistent with previous findings.

Tumor microenvironment was involved in the progression of various types of tumors [28, 29]. The exploration of novel therapeutic targets which contributed to remodeling of tumor microenvironment was very important for OC patients [30, 31]. To delve into the involvements of RIPK4 expressions with immune microenvironment, we performed the CIBERSORT algorithm. Moreover, we observed that two kinds of tumor-infiltrating immune cells were positively correlated with RIPK4 expression, including monocytes and T cell CD4 memory resting; five kinds of TICs were negatively correlated with RIPK4 expressions, including T cell gamma delta, T cell follicular helper, T cell CD8, T cell CD4 memory activated, and Macrophages M1. Our findings revealed that the dysregulation of RIPK4 may influence the function of tumor microenvironment.

There were some limitations to our analysis. First, this study was based merely on the sequencing data and clinical data from TCGA datasets and GEO datasets. More studies with different populations with more patients are necessary to demonstrate our findings. Second, there was a lack of in vitro and in vivo assays, which are crucial to demonstrate the potential functions of RIPK4 in OC.

## 5. Conclusion

Our findings uncovered a novel OC-related gene, RIPK4 which may be used as a therapeutic target or predictive marker for OC patients.

## Data Availability

The data used to support the findings of this study are available from the corresponding authors upon request.

## Supplementary Materials

Supplementary Materials Table S1: list of TCGA cancer types.
