# Supplementary material for: RIPK4 Is an Immune Regulating-Associated Biomarker for Ovarian Cancer and Possesses Generalization Value in Pan-Cancer
Source: J Immunol Res. 2022 Mar 9;2022:7599098. doi: 10.1155/2022/7599098 (PMC8926548; doi:10.1155/2022/7599098)
Supplement: Supplementary Materials — Table S1: list of TCGA cancer types. [file 7599098.f1.docx]

**Table S1: List of TCGA cancer types.**

| Project(Abbreviations) | Cancer Type | Primary Site |
| --- | --- | --- |
| BLCA | Bladder Urothelial Carcinoma | Bladder |
| BRCA | Breast Invasive Carcinoma | Breast |
| CESC | Cervical Squamous Cell Carcinoma and Endocervical Adenocarcinoma | Cervix |
| CHOL | Cholangiocarcinoma | Bile Duct |
| CRC | Colorectal Cancer | Colorectal |
| ESCA | Esophageal Carcinoma | Esophagus |
| GBM | Glioblastoma Multiforme | Brain |
| HNSC | Head and Neck Squamous Cell Carcinoma | Head and Neck |
| KICH | Kidney Chromophobe | Kidney |
| KIRC | Kidney Renal Clear Cell Carcinoma | Kidney |
| KIRP | Kidney Renal Papillary Cell Carcinoma | Kidney |
| LIHC | Hepatocellular Carcinoma | Liver |
| LUAD | Lung Adenocarcinoma | Lung |
| LUSC | Lung Squamous Cell Carcinoma | Lung |
| PAAD | Pancreatic Adenocarcinoma | Pancreas |
| PCPG | Pheochromocytoma and Paraganglioma | Adrenal Gland |
| PRAD | Prostate Adenocarcinoma | Prostate |
| STAD | Stomach Adenocarcinoma | Stomach |
| THCA | Thyroid Carcinoma | Thyroid |
| UCEC | Uterine Corpus Endometrial Carcinoma | Uterus |
